# Supplementary material for: Developing RT-LAMP assays for rapid diagnosis of SARS-CoV-2 in saliva
Source: eBioMedicine. 2021 Dec 16;75:103736. doi: 10.1016/j.ebiom.2021.103736 (PMC8674011; doi:10.1016/j.ebiom.2021.103736)
Supplement: Supplementary file 1 [file mmc1.pdf]

Table S1. The N Gene primer sets

Primer sets	Amplifcation Len	Sequence (5'-3')			Sequence (5'-3')			Sequence (5'-3')			Sequence (5'-3')			Sequence (5'-3')		
#	B2-F2	F3	Len	Tm	F2	Len	Tm	FL	Len	Tm	F1c	Len	Tm	B1c	Len	Tm
N1	242	atcacattggcacccgcaatc	21	63.6	gctgcaatcgtgctacaacttc	22	62.3	cttttggcaatgttgttccttga	23	60.1	ctctgctcccttctgcgtaga	21	63	tgatgctgctcttgctttgctg	22	63.9
N1(-3)	242										tgctcccttctgcgtaga	18	59.7	gctgctcttgctttgctg	18	59.5
N1(-6)	242										tcccttctgcgtaga	15	51.7	gctcttgctttgctg	15	51.9
N1(-9)	242										cttctgcgtaga	12	39.4	cttgctttgctg	12	41.1
N2	146	gaattcgtggtggtgacggtaa	22	62.5	attcaaggctccctcagttgc	21	62.4	ccgtctttgttagcaccatagg	22	60.7	attcaaggctccctcagttgc	21	62.4	atcacattggcacccgcaatc	21	63.6
N2(-5)	146				aggctccctcagttgc	16	57.7				aggctccctcagttgc	16	57.7	attggcacccgcaatc	16	57.1
N3	149	cccgcattacgtttggtggac	21	63.8	agaatggagaacgcagtggg	20	61.9	gccgacgttgttttgatcgc	20	62.8	cagtattattgggtaaacctt	21	52.5	atggcaaggaagacct	16	53.6
N3(-2)	149										gtattattgggtaaacctt	19	48.8	ggcaaggaagacct	14	49.8
N3(-4)	149										attattgggtaaacctt	17	46	caaggaagacct	12	38.8
N3(-6)	149										tattgggtaaacctt	15	42.8	aggaagacct	10	29.7
N4	142	taggaactgggccagaagctg	21	63.6	gtgctaacaaagacggcatcat	22	61.2	tattcaaggctccctcagttgc	22	62	ggtgccaatgtgatctt	17	53.9	ctgctaacaatgctgc	16	52.4
N4(-2)	142										tgccaatgtgatctt	15	47.9	gctaacaatgctgc	14	47.7
N4(-4)	142										ccaatgtgatctt	13	38.8	taacaatgctgc	12	38.7
N4(-6)	142										aatgtgatctt	11	27.5	acaatgctgc	10	34.7
N5	163	gtgctaacaaagacggcatcat	22	61.2	tgctaacaatgctgcaatcgtg	22	62	ttggcaatgttgttccttgagg	22	61.3	ctcccttctgcgtaga	16	53.6	acgtagtcgcaacag	15	52.3
N5(-2)	163										cccttctgcgtaga	14	49.3	gtagtcgcaacag	13	44.6
N5(-4)	163										cttctgcgtaga	12	39.8	agtcgcaacag	11	39.8
N5(-6)	163										tctgcgtaga	10	32.2	tcgcaacag	9	29.9
N6	137	aacaatgctgcaatcgtgctac	22	62	gtcaagcctcttctcgttcctc	22	62.3	ttgaactgttgcgactacgtga	22	62.1	ctgcctggagttgaat	16	52.3	cctgctagaatggctg	16	53.1
N6(-2)	137										gcctggagttgaat	14	47.3	tgctagaatggctg	14	47.1
N6(-4)	137										ctggagttgaat	12	35.9	ctagaatggctg	12	37.3
N6(-6)	137										ggagttgaat	10	25.8	agaatggctg	10	31
N7	143	taggaactgggccagaagctg	21	63.6	gtgctaacaaagacggcatcat	22	61.2	tattcaaggctccctcagttgc	22	62	tgccaatgtgatctt	15	47.9	aatcgtgctacaac	14	45.2
N8	154	gcaactgagggagccttgaata	22	62	aacaatgctgcaatcgtgctac	22	62	ttggcaatgttgttccttgagg	22	61.3	tcccttctgcgta	13	46.7	gtcgcaacagttc	13	45.9
N9	119	ttctacgcagaagggagcagag	22	63.4	aaattcaactccaggcagcagt	22	62.4	gccattctagcaggagaagttcc	23	62.5	catcaccgccatt	13	47.2	ttgctttgctgct	13	47.2
N10	140	cctcagattcaactggcagtaac	23	60.9	taatactgcgtcttggttcacc	22	60	tccttgccatgttgagtgagag	22	61.9	gccttgtcctcga	13	49	caatagcagtccaga	15	47.4
N11	139	taggaactgggccagaagctg	21	63.6	tgcaactgagggagccttgaat	22	63.9	gtgccaatgtgatcttttggtg	22	60.3	ttgttagcaggattg	15	45.7	caaggaacaacattg	15	45.2
N12	138	taggaactgggccagaagctg	21	63.6	agggagccttgaatacaccaaa	22	61.3	gattgcgggtgccaatgtgat	21	63.6	gcacgattgcag	12	45.1	acaacattgccaaa	14	45.2
N13	144	cttccctatggtgctaacaaagac	24	60.8	cccgcaatcctgctaacaatg	21	61.6	cttgaggaagttgtagcacgat	22	60	ccttttggcaatgtt	15	47.6	agtcaagcctcttc	14	46.4
N14	158	ggtgctaacaaagacggcatca	22	63.1	ctgctaacaatgctgcaatcgt	22	61.8	ttggcaatgttgttccttgagg	22	61.3	ccttctgcgtagaa	14	46.7	tcatcacgtagtcg	14	46.7
N15	158	gcaactgagggagccttgaata	22	62	acaatgctgcaatcgtgctaca	22	63.1	ttggcaatgttgttccttgagg	22	61.3	ccttctgcgtagaa	14	46.7	acgtagtcgcaac	13	47.1
N16	126	tgcaactgagggagccttgaat	22	63.9	tgctgcaatcgtgctacaactt	22	63	ttttggcaatgttgttccttgag	23	60.1	ccttctgcgtagaa	14	46.7	agtcaagcctcttc	14	46.4
N17	138	gggagccttgaatacaccaaaa	22	60.5	cctcaaggaacaacattgccaa	22	61.3	cctctgctcccttctgcgtag	21	64	aacgagaagaggc	13	45.7	tcgcaacagttca	13	45.9
N18	159	taacaatgctgcaatcgtgcta	22	60.6	cagtcaagcctcttctcgttcc	22	62.6	gaactgttgcgactacgtgatg	22	61.5	gcctggagttgaat	14	47.3	ctgctcttgctttg	14	47
N19	163	aacaatgctgcaatcgtgctac	22	62	gtcaagcctcttctcgttcctc	22	62.3	ttgaactgttgcgactacgtga	22	62.1	gcctggagttgaat	14	47.3	ctgctcttgctttg	14	47
N20	145	cctcaaggaacaacattgccaa	22	61.3	tctcgttcctcatcacgtagtc	22	60.9	tgcctggagttgaatttcttgaa	23	60.4	caggagaagttccc	14	47	tgatgctgctcttg	14	48
N21	143	ctcaaggaacaacattgccaaaa	23	60.1	cctcatcacgtagtcgcaacag	22	62.9	gctgcctggagttgaatttctt	22	61.1	ctagcaggagaagt	14	45.1	ctgctcttgctttg	14	47
N22	119	ttctacgcagaagggagcagag	22	63.4	aattcaactccaggcagcagta	22	61.6	gccattctagcaggagaagttcc	23	62.5	catcaccgccat	12	45.2	ttgctttgctgct	13	47.2
N23	149	ttctacgcagaagggagcagag	22	63.4	tcaactccaggcagcagtagg	21	63.5	gccattctagcaggagaagttc	22	60.4	tcaccgccattg	12	45.7	gcaaaatgtctggt	14	45.6
N24	146	ttctacgcagaagggagcagag	22	63.4	caactccaggcagcagtagg	20	62.2	gccattctagcaggagaagttc	22	60.4	catcaccgccatt	13	47.2	gcaaaatgtctggt	14	45.6
N25	122	cagtcaagcctcttctcgttcc	22	62.6	cctgctagaatggctggcaatg	22	63.6	caaagcaagagcagcatcacc	21	62.1	tcaatctgtcaagca	15	47.5	atgtctggtaaaggc	15	48.1
N26	142	ccgcattacgtttggtggacc	21	63.8	agaatggagaacgcagtggg	20	61.9	gccgacgttgttttgatcgc	20	62.8	attattgggtaaacctt	17	46	ggcaaggaagacc	13	47.1
N27	143	ccctcagattcaactggcagta	22	61.4	gggcgcgatcaaaacaacg	19	62.7	gacgcagtattattgggtaaacc	24	60.4	gagagcggtgaac	13	47.6	tcgaggacaaggc	13	49
N28	125	ccgcattacgtttggtggacc	21	63.8	cagaatggagaacgcagtggg	21	63	gccgacgttgttttgatcgc	20	62.8	gcagtattattgggta	16	45.4	ttcaccgctctca	13	47.7

Primer sets	Sequence (5'-3')			Sequence (5'-3')			Sequence (5'-3')			Sequence (5'-3')		Sequence (5'-3')		PubMed
#	BL	Len	Tm	B2	Len	Tm	B3	Len	Tm	FIP(F1c+F2)	Len	BIP(B1c+B2)	Len	Accession
N1	tgaaccagcttgagagcaaaat	22	60.4	ttgttgttgttggcctttacca	22	60.8	acattgtatgctttagtggcagt	24	60	ctctgctcccttctgcgtagatcggctgcaatcgtgctacaactt	46	tgatgctgctcttgctttgctggacttgttgttgttggccttta	47	NC_045512
N1(-3)										tgctcccttctgcgtagatcggctgcaatcgtgctacaacttc	43	gctgctcttgctttgctggacttgttgttgttggcctttacca	43	NC_045512
N1(-6)										tcccttctgcgtagatcggctgcaatcgtgctacaacttc	40	gctcttgctttgctggacttgttgttgttggcctttacca	40	NC_045512
N1(-9)										cttctgcgtagatcggctgcaatcgtgctacaacttc	37	cttgctttgctggacttgttgttgttggcctttacca	37	NC_045512
N2	gcaatcgtgctacaacttcctc	22	61.5	atcacattggcacccgcaatc	21	63.6	aactgttgcgactacgtgatga	22	61.9	attcaaggctccctcagttgcactgggccagaagctggactt	42	atcacattggcacccgcaatcagccttttggcaatgttgttcc	43	NC_045512
N2(-5)				attggcacccgcaatc	16	57.1				aggctccctcagttgcactgggccagaagctggactt		attggcacccgcaatcagccttttggcaatgttgttcc	38	NC_045512
N3	aggacaaggcgttccaattaac	22	60.8	caatttggtcatctggactgct	22	60.6	ttttaccgtcaccaccacgaat	22	62.1	cagtattattgggtaaaccttagaatggagaacgcagtggg	41	atggcaaggaagacctcaatttggtcatctggactgct	38	NC_045512
N3(-2)										gtattattgggtaaaccttagaatggagaacgcagtggg	39	ggcaaggaagacctcaatttggtcatctggactgct	36	NC_045512
N3(-4)										attattgggtaaaccttagaatggagaacgcagtggg	37	caaggaagacctcaatttggtcatctggactgct	34	NC_045512
N3(-6)										tattgggtaaaccttagaatggagaacgcagtggg	35	aggaagacctcaatttggtcatctggactgct	32	NC_045512
N4	tcaaggaacaacattgccaaaag	23	60.1	cctctgctcccttctgcgtag	21	64	aggaacgagaagaggcttgact	22	62.8	ggtgccaatgtgatcttgtgctaacaaagacggcatcat	39	ctgctaacaatgctgccctctgctcccttctgcgtag	37	NC_045512
N4(-2)										tgccaatgtgatcttgtgctaacaaagacggcatcat	37	gctaacaatgctgccctctgctcccttctgcgtag	35	NC_045512
N4(-4)										ccaatgtgatcttgtgctaacaaagacggcatcat	35	taacaatgctgccctctgctcccttctgcgtag	33	NC_045512
N4(-6)										aatgtgatcttgtgctaacaaagacggcatcat	33	acaatgctgccctctgctcccttctgcgtag	31	NC_045512
N5	aactccaggcagcagtaggg	20	63.4	gccagccattctagcaggaga	21	63.7	aagctggttcaatctgtcaagc	22	61.1	ctcccttctgcgtagatgctaacaatgctgcaatcgtg	38	acgtagtcgcaacaggccagccattctagcaggaga	36	NC_045512
N5(-2)										cccttctgcgtagatgctaacaatgctgcaatcgtg	36	gtagtcgcaacaggccagccattctagcaggaga	34	NC_045512
N5(-4)										cttctgcgtagatgctaacaatgctgcaatcgtg	34	agtcgcaacaggccagccattctagcaggaga	32	NC_045512
N5(-6)										tctgcgtagatgctaacaatgctgcaatcgtg	32	tcgcaacaggccagccattctagcaggaga	30	NC_045512
N6	atgctgctcttgctttgctg	20	61.6	tgctctcaagctggttcaatct	22	61.6	gtttggccttgttgttgttggc	22	63.4	ctgcctggagttgaatgtcaagcctcttctcgttcctc	38	cctgctagaatggctgtgctctcaagctggttcaatct	38	NC_045512
N6(-2)										gcctggagttgaatgtcaagcctcttctcgttcctc	36	tgctagaatggctgtgctctcaagctggttcaatct	36	NC_045512
N6(-4)										ctggagttgaatgtcaagcctcttctcgttcctc	35	ctagaatggctgtgctctcaagctggttcaatct	35	NC_045512
N6(-6)										ggagttgaatgtcaagcctcttctcgttcctc	33	agaatggctgtgctctcaagctggttcaatct	33	NC_045512
N7	tcaaggaacaacattgccaaaag	23	60.1	cctctgctcccttctgcgta	20	63.2	gaggaacgagaagaggcttgac	22	62.3	tgccaatgtgatcttgtgctaacaaagacggcatcat	37	aatcgtgctacaaccctctgctcccttctgcgta	34	NC_045512
N8	aattcaactccaggcagcagta	22	61.6	gccattctagcaggagaagttc	22	60.4	aagctggttcaatctgtcaagc	22	61.1	tcccttctgcgtaaacaatgctgcaatcgtgctac	35	gtcgcaacagttcgccattctagcaggagaagttc	35	NC_045512
N9	gacagattgaaccagcttgaga	22	60	tgttgttgttggcctttaccag	22	61.2	gtatgctttagtggcagtacgtt	23	60.6	catcaccgccattaaattcaactccaggcagcagt	35	ttgctttgctgcttgttgttgttggcctttaccag	35	NC_045512
N10	ctaccgaagagctaccagacga	22	62.6	attttaccgtcaccaccacgaa	22	62.1	cagcttctggcccagttccta	21	63.6	gccttgtcctcgataatactgcgtcttggttcacc	35	caatagcagtccagaattttaccgtcaccaccacgaa	37	NC_045512
N11	acgcagaagggagcagagg	19	63.6	aggaacgagaagaggcttgact	22	62.8	ttcttgaactgttgcgactacg	22	60.8	ttgttagcaggattgtgcaactgagggagccttgaat	37	caaggaacaacattgaggaacgagaagaggcttgact	37	NC_045512
N12	acgcagaagggagcagagg	19	63.6	cgtgatgaggaacgagaagagg	22	62.1	gctgcctggagttgaatttcttg	23	62.2	gcacgattgcagagggagccttgaatacaccaaa	34	acaacattgccaaacgtgatgaggaacgagaagagg	36	NC_045512
N13	catcacgtagtcgcaacagttc	22	61.5	ctactgctgcctggagttgaat	22	62	aagctggttcaatctgtcaagc	22	61.1	ccttttggcaatgttcccgcaatcctgctaacaatg	36	agtcaagcctcttcctactgctgcctggagttgaat	36	NC_045512
N14	aattcaactccaggcagcagta	22	61.6	gccattctagcaggagaagttcc	23	62.5	aagctggttcaatctgtcaagc	22	61.1	ccttctgcgtagaactgctaacaatgctgcaatcgt	36	tcatcacgtagtcggccattctagcaggagaagttcc	37	NC_045512
N15	caactccaggcagcagtagg	20	62.2	gccagccattctagcaggaga	21	63.7	gttggcctttaccagacattttg	23	60.2	ccttctgcgtagaaacaatgctgcaatcgtgctaca	36	acgtagtcgcaacgccagccattctagcaggaga	34	NC_045512
N16	catcacgtagtcgcaacagttc	22	61.5	cctactgctgcctggagttgaa	22	63.9	cagcaaagcaagagcagcat	20	61.6	ccttctgcgtagaatgctgcaatcgtgctacaactt	36	agtcaagcctcttccctactgctgcctggagttgaa	36	NC_045512
N17	aattcaactccaggcagcagta	22	61.6	gccattgccagccattctagc	21	64	aagctggttcaatctgtcaagc	22	61.1	aacgagaagaggccctcaaggaacaacattgccaa	35	tcgcaacagttcagccattgccagccattctagc	34	NC_045512
N18	ttgacagattgaaccagcttgag	23	60.7	gttggcctttaccagacattttg	23	60.2	cagcagcagatttcttagtgaca	23	60.5	gcctggagttgaatcagtcaagcctcttctcgttcc	36	ctgctcttgctttggttggcctttaccagacattttg	37	NC_045512
N19	ttgacagattgaaccagcttgag	23	60.7	ttgttgttggcctttaccagac	22	61	atgctttagtggcagtacgttt	22	60.2	gcctggagttgaatgtcaagcctcttctcgttcctc	36	ctgctcttgctttgttgttgttggcctttaccagac	36	NC_045512
N20	gcttgacagattgaaccagctt	22	61.1	tggcctttaccagacattttgc	22	61.3	gcagcagatttcttagtgacagt	24	61	caggagaagttccctctcgttcctcatcacgtagtc	36	tgatgctgctcttgtggcctttaccagacattttgc	36	NC_045512
N21	gcttgacagattgaaccagctt	22	61.1	gttgttggcctttaccagacat	22	60.8	atgctttagtggcagtacgttt	22	60.2	ctagcaggagaagtcctcatcacgtagtcgcaacag	36	ctgctcttgctttggttgttggcctttaccagacat	36	NC_045512
N22	gacagattgaaccagcttgaga	22	60	ttgttgttgttggcctttacca	22	60.8	gtatgctttagtggcagtacgtt	23	60.6	catcaccgccataattcaactccaggcagcagta	34	ttgctttgctgctttgttgttgttggcctttacca	35	NC_045512
N23	gccaacaacaacaaggccaaac	22	63.4	cctcagcagcagatttcttagtg	23	60.9	gtatgctttagtggcagtacgtt	23	60.6	tcaccgccattgtcaactccaggcagcagtagg	33	gcaaaatgtctggtcctcagcagcagatttcttagtg	37	NC_045512
N24	gccaacaacaacaaggccaaac	22	63.4	tcagcagcagatttcttagtgac	23	60.3	gtatgctttagtggcagtacgtt	23	60.6	catcaccgccattcaactccaggcagcagtagg	33	gcaaaatgtctggttcagcagcagatttcttagtgac	37	NC_045512
N25	caacaacaaggccaaactgtca	22	61.7	agcctcagcagcagatttctta	22	61.4	ggaccacgtctgccgaaag	19	62.9	tcaatctgtcaagcacctgctagaatggctggcaatg	37	atgtctggtaaaggcagcctcagcagcagatttctta	37	NC_045512
N26	cgaggacaaggcgttccaatta	22	62.6	gtcatctggactgctattggtg	22	60.4	ttcgtctggtagctcttcggta	22	62.3	attattgggtaaaccttagaatggagaacgcagtggg	37	ggcaaggaagaccgtcatctggactgctattggtg	35	NC_045512
N27	ccaattaacaccaatagcagtcca	24	60.8	ttcggtagtagccaatttggtc	22	60	ttcattttaccgtcaccaccac	22	60.8	gagagcggtgaacgggcgcgatcaaaacaacg	32	tcgaggacaaggcttcggtagtagccaatttggtc	35	NC_045512
N28	gcaaggaagaccttaaattccct	23	60.2	ggtgttaattggaacgccttgt	22	61.3	gtctggtagctcttcggtagta	22	60.1	gcagtattattgggtacagaatggagaacgcagtggg	37	ttcaccgctctcaggtgttaattggaacgccttgt	35	NC_045512

Note: Each primer set is composed of eight oligos, in which FIP is made by F1c+F2, and BIP is made by B1c+B2. Len, Length.

Table S2. N6 assay false-positive product sequencing

**Primers to establish library**	**Forward**	AATGATACGGCGACCACCGAGATCTACAC TGCACGGTAACACTCTTTCCCTACACGACGCTCTTCCGATCTGTCAAGCCTCTTCTCGTTCCTC	index in red				underscore incates the F2 of N6
	**Reverse**	CAAGCAGAAGACGGCATACGAGATTGCACGGTAGTGACTGGAGTTCAGACGTGTGCTCTTCCGATGCTCTCAAGCTGGTTCAATCT	index in red				underscore incates the B2 of N6
**Sequencing results**		**Top 10 reads**					
		GTCAATCCTCTTCTCGTTCCTCCTGCTAGATTGAACCAGCTTGAGAGCA		2120402	35.36%		
		GTCAATCCTCTTCTCGTTCCTCCTGCTAGATTGAACCAGCTTGAGAGCAA		66114	1.10%	36.46%	
		GTCAATCCTCTTCTCGTTCCTCCTGCTAGATTGAACCAGCTTGAGAGCAAAGCCATTCAAGCAGGGCTGCCTGGAGTTGAATGTCAAGCCTCTTCTCGTTCCTCCTGCTAGATTTAACCACCTTGAGCGCA		1432573	23.89%	23.89%	
		GTCAATCCTCTTCTCGTTCCTCCTGCTAGATTGAACCAGCTTGAGAGCAAAGCCATTCAAGCAGGGCTGCCTGGAGTTGAATGTCAAGCCTCTTCTCGTTCCTCCTGCTAGATTTAACCACCTTGAGCGCACCGCCAGTCAAGCAGGGATC		917862	15.31%		
		GTCAATCCTCTTCTCGTTCCTCCTGCTAGATTGAACCAGCTTGAGAGCAAAGCCATTCAAGCAGGGCTGCCTGGAGTTGAATGTCAAGCCTCTTCTCGTTCCTCCTGCTAGATTTAACCACCTTGAGCGCACCGCCAGTCAAGCAGGGAT		217254	3.62%		
		GTCAATCCTCTTCTCGTTCCTCCTGCTAGATTGAACCAGCTTGAGAGCAAAGCCATTCAAGCAGGGCTGCCTGGAGTTGAATGTCAAGCCTCTTCTCGTTCCTCCTGCTAGATTTAACCACCTTGAGCGCACCGCCAGTCAAGCAGGGA		70644	1.18%		
		GTCAATCCTCTTCTCGTTCCTCCTGCTAGATTGAACCAGCTTGAGAGCAAAGCCATTCAAGCAGGGCTGCCTGGAGTTGAATGTCAAGCCTCTTCTCGTTCCTCCTGCTAGATTTAACCACCTTGAGCGCACCGCCAGTC		55081	0.92%		
		GTCAATCCTCTTCTCGTTCCTCCTGCTAGATTGAACCAGCTTGAGAGCAAAGCCATTCAAGCAGGGCTGCCTGGAGTTGAATGTCAAGCCTCTTCTCGTTCCTCCTGCTAGATTTAACCACCTTGAGCGCACCGCCAGTCAAGC		31607	0.53%	22.36%	
		GTCAATCCTCTTCTCGTTCCTCCAGGCAGCCTGCTAGATTGAACCAGCTTGAGAGCA		48189	0.80%		
		GTCAATCCTCTTCTCGTTCCTCCTGCTAGATTGAACCAGCTTGAGAGCAAAGCCATTCAAGCAGGGCTGCCTGGAGT		27643	0.46%		
			**Total**	**5996593**		**83.17%**	

Table S3. The loop stem (F1c and B1c), LAMP amplification efficiency, and specificity

primer sets	F1c	Length	Tm (°C)	B1c	Length	Tm(°C)	TT (min)	FP
**N geme**							**LAMP**	
N7	tgccaatgtgatctt	15	47.9	aatcgtgctacaac	14	45.2	19.7±0.8	1
N8	tcccttctgcgta	13	46.7	gtcgcaacagttc	13	45.9	43.9±5.3	3
N9	catcaccgccatt	13	47.2	ttgctttgctgct	13	47.2	24.4±1.7	2
N10	gccttgtcctcga	13	49	caatagcagtccaga	15	47.4	28.6±3.1	1
N11	ttgttagcaggattg	15	45.7	caaggaacaacattg	15	45.2	28.4±6.6	1
N12	gcacgattgcag	12	45.1	acaacattgccaaa	14	45.2	32.9±7.1	1
N13	ccttttggcaatgtt	15	47.6	agtcaagcctcttc	14	46.4	26.0±3.5	3
N14	ccttctgcgtagaa	14	46.7	tcatcacgtagtcg	14	46.7	27.5±3.0	3
N15	ccttctgcgtagaa	14	46.7	acgtagtcgcaac	13	47.1	26.6±2.0	2
N16	ccttctgcgtagaa	14	46.7	agtcaagcctcttc	14	46.4	25.7±2.3	2
N17	aacgagaagaggc	13	45.4	tcgcaacagttca	13	45.9	30.5±3.3	1
N18	gcctggagttgaat	14	47.3	ctgctcttgctttg	14	47	35.2±1.7	2
N19	gcctggagttgaat	14	47.3	ctgctcttgctttg	14	47	31.2±3.5	2
N20	caggagaagttccc	14	47	tgatgctgctcttg	14	48	27.4±3.1	2
N21	ctagcaggagaagt	14	45.1	ctgctcttgctttg	14	47	42.6±4.9	2
N22	catcaccgccat	12	45.2	ttgctttgctgct	13	47.2	41.7±4.4	2
N23	tcaccgccattg	12	45.7	gcaaaatgtctggt	14	45.6	22.6±2.4	2
N24*	catcaccgccatt	13	47.2	gcaaaatgtctggt	14	45.6	21.6±2.6	1
N25*	tcaatctgtcaagca	15	47.5	atgtctggtaaaggc	15	48.1	18.0±3.9	1
N26	attattgggtaaacctt	17	46	ggcaaggaagacc	13	47.1	34.9±6.6	1
N27*	gagagcggtgaac	13	47.6	tcgaggacaaggc	13	49	18.4±1.8	1
N28	gcagtattattgggta	16	45.4	ttcaccgctctca	13	47.7	27.5±6.7	1
							**RT-LAMP**	
N2(-5)	aggctccctcagttgc	16	57.7	attggcacccgcaatc	16	57.1	15.3±0.3	1
N3(-4)	attattgggtaaacctt	17	46	caaggaagacct	12	38.8	23.1±3.2	1
N4(-4)	ccaatgtgatctt	13	38.8	taacaatgctgc	12	38.7	21.4±4.9	1
N6(-4)	ctggagttgaat	12	35.9	ctagaatggctg	12	35.9	21.9±3.4	1
N7	tgccaatgtgatctt	15	47.9	aatcgtgctacaac	14	45.2	14.3±0.6	1
N24*							20.4±1.8	3
N25*							20.2±5.5	1
N27*							11.7±0.1	1
**M gene**								
M1	tctttaggcaggtc	14	45.9	actgttgctacatca	15	47.1	22.6±2.9	1
M2	gtccttgatgtcac	14	45.1	caaattgggagcttc	15	47.6	22.0±0.9	2
M3	cagaatagtgccatg	15	46.6	agaaagtgaactcgt	15	47.1	11.1±0.8	1
M4	gcaggtccttgat	13	45.4	ggagcttcgcag	14	46.8	31.1±2.3	1
M5	aaacagtctgaaagaa	16	45.7	ttctcaacgtgcc	13	47.2	16.5±0.4	1
M6	acatggaacgcg	12	46.6	ttcttctcaacgtgc	15	49.6	15.2±0.4	1
**S gene**								
S1	gcaaaggtggca	12	46.4	tgattgctcaatacac	16	47.1	24.8±2.1	1
S2	aggccgttaaactt	14	46.6	gttttgccaccttt	14	46.3	15.5±1.4	3
S3	gaggtctctagcag	14	46	gtttaacggccttac	15	47.4	26.1±4.9	3
S4	gcaaaggtggcaaa	14	49.9	tgattgctcaatacac	16	47.1	23.1±2.9	1
S5	gtgtctgtggatca	14	46.5	acaccaggaacaaa	14	46.2	21.5±1.8	4
S6	tcaagtgtctgtgg	14	46.8	caccaggaacaaata	15	45.1	17.5±0.8	4
S7	tgaggtctctagca	14	46.5	tttaacggccttact	15	47.1	19.2±2.9	1

TT represented 3-10 independent measures presented with mean± SD. * same primer sets were used both for LAMP and RT-LAMP assays.
FP was determined as described in "Method". From 3-4 replicates, the highest FP score was listed as the final FP for the primer.

Table S4. The M Gene primer sets

Primer sets	Amplifcation Len	Sequence (5'-3')			Sequence (5'-3')			Sequence (5'-3')		
**#**	**B2-F2(Length)**	**F3**	**Len**	**Tm**	**F2**	**Len**	**Tm**	**FL**	**Len**	**Tm**
**M1**	123	actattctgaccagaccgcttc	22	62	ttcgtggacatcttcgtattgc	22	60.7	gatgtcacagcgtcctagatgg	22	62
**M2**	157	cattcttcctcaacgtgccactc	22	61	cggagctgtgatccttcgtg	20	62.6	gtgtccagcaatacgaagatgt	22	60
**M3**	129	gctatcgcaatggcttgtcttg	22	62	ccatgtggtcattcaatccaga	22	60	ggcacgttgagaagaatgttagt	23	61
**M4**	166	cattcttctcaacgtgccactc	22	61	tgtgatccttcgtggacatctt	22	61.1	tcctagatggtgtccagcaatac	23	61
**M5**	160	ggccagtaactttagcttgttttg	24	61	cgcaatggcttgtcttgtagg	21	61.6	tgaagtagctgagccacatcaa	22	61
**M6**	141	gttttgtgcttgctgctgttta	22	60	aggcttgatgtggctcagcta	21	63.3	gcaaacagtctgaaagaagcaatg	24	61

Primer sets	Sequence (5'-3')			Sequence (5'-3')			Sequence (5'-3')		
**#**	**F1c**	**Len**	**Tm**	**B1c**	**Len**	**Tm**	**BL**	**Len**	**Tm**
**M1**	tctttaggcaggtc	14	46	actgttgctacatca	15	47.1	cttattacaaattgggagcttcgc	24	60
**M2**	gtccttgatgtcac	14	45	caaattgggagcttc	15	47.6	gcgtgtagcaggtgactcag	20	63
**M3**	cagaatagtgccatg	15	47	agaaagtgaactcgt	15	47.1	gatccttcgtggacatcttcgt	22	62
**M4**	gcaggtccttgat	13	45	ggagcttcgcag	12	46.8	ggtgactcaggttttgctgcat	22	63
**M5**	aaacagtctgaaagaa	16	46	ttctcaacgtgcc	13	47.2	tattctgaccagaccgcttcta	22	60
**M6**	acatggaacgcg	12	47	ttcttctcaacgtgc	15	49.6	tattctgaccagaccgcttcta	22	60

Primer sets	Sequence (5'-3')			Sequence (5'-3')		
**#**	**B2**	**Len**	**Tm**	**B3**	**Len**	**Tm**
**M1**	aaacctgagtcacctgctacac	22	62	attgtcactgctactggaatgg	22	60
**M2**	tgtagcgactgtatgcagcaaa	22	62	gctactggaatggtctgtgttt	22	60.2
**M3**	cgtcctagatggtgtccagcaa	22	64	gaaagcgttcgtgatgtagcaa	22	61.4
**M4**	ttatagttgccaatcctgtagcg	23	60	gctactgggaatggtctgtgttt	22	60.2
**M5**	cagctccgattacgagttcact	22	62	gtccagcaatacgaagatgtcc	22	60.7
**M6**	agctccgattacgagttcactt	22	61	gtccagcaatacgaagatgtcc	22	60.7

Primer sets	Sequence (5'-3')		Sequence (5'-3')		PubMed
**#**	**FIP(F1c+F2)**	**Len**	**BIP(B1c+B2)**	**Len**	**Accession**
**M1**	tctttaggcaggtcttcgtggacatcttcgtattgc	36	actgttgctacatcaaaacctgagtcacctgctacac	37	NC_045512
**M2**	gtccttgatgtcaccggagctgtgatccttcgtg	34	caaattgggagcttctgtagcgactgtatgcagcaaa	37	NC_045512
**M3**	cagaatagtgccatgccatgtggtcattcaatccaga	37	agaaagtgaactcgtcgtcctagatggtgtccagcaa	37	NC_045512
**M4**	gcaggtccttgattgtgatccttcgtggacatctt	35	ggagcttcgcagttatagttgccaatcctgtagcg	35	NC_045512
**M5**	aaacagtctgaaagaacgcaatggcttgtcttgtagg	37	ttctcaacgtgcccagctccgattacgagttcact	35	NC_045512
**M6**	acatggaacgcgaggcttgatgtggctcagcta	33	ttcttctcaacgtgcagctccgattacgagttcactt	37	NC_045512

*** Note: Each primer set is composed of eight oligos, in which FIP is made by F1c+F2, and BIP is made by B1c+B2.**

Table S5. The S Gene primer sets

Primer sets #	Amplifcation Len B2-F2(Len)	Sequence (5'-3') F3	Len	Tm	Sequence (5'-3') F2	Len	Tm	Sequence (5'-3') FL	len	Tm
S1	124	acacttgcagatgctggctt	20	62.4	tattgctgctagagacctcattt	23	59	agtaaggccgttaaacttttgtg	23	59.4
S2	122	agtgacacttgcagatgctg	20	60.3	aatatggtgattgccttggtga	22	59.7	gcacaaatgaggtctctagcag	22	60.7
S3	140	attaccagatccatcaaaacca	22	57.1	agtgacacttgcagatgctg	20	60.3	accaaggcaatcaccatattgt	22	60
S4	133	agtgacacttgcagatgctg	20	60.3	cttggtgatattgctgctagag	22	58.3	gtaaggccgttaaacttttgtg	22	58.1
S5	174	tcaatggtttaacaggcacagg	22	60.8	aagtttctgcctttccaacaat	22	58.6	cagtagtgtcagcaatgtctct	22	59.4
S6	169	tcaatggtttaacaggcacagg	22	60.8	ctgcctttccaacaatttggc	21	60.6	tcagtagtgtcagcaatgtctc	22	59.4
S7	136	attaccagatccatcaaaacca	22	57.1	cacttgcagatgctggctt	19	60.7	accaaggcaatcaccatattgt	22	60

Primer sets #	Sequence (5'-3') F1c	Len	Tm	Sequence (5'-3') B1c	Len	Tm	Sequence (5'-3') BL	Len	Tm
S1	gcaaaggtggca	12	46.4	tgattgctcaatacac	16	47.1	ctgttagcgggtacaatcactt	22	60
S2	aggccgttaaactt	14	46.6	gttttgccaccttt	14	46.3	cacagatgaaatgattgctcaa	22	57
S3	gaggtctctagcag	14	46	gtttaacggccttac	15	47.4	cctttgctcacagatgaaatga	22	58.6
S4	gcaaaggtggcaaa	14	49.9	tgattgctcaatacac	16	47.1	gttagcgggtacaatcacttct	22	59.8
S5	gtgtctgtggatca	14	46.5	acaccaggaacaaa	14	46.2	taaccaggttgctgttctttat	22	57.3
S6	tcaagtgtctgtgg	14	46.8	caccaggaacaaata	15	45.1	aaccaggttgctgttctttatc	22	58.5
S7	tgaggtctctagca	14	46.5	tttaacggccttact	15	47.1	cctttgctcacagatgaaatga	22	58.6

Primer sets #	Sequence (5'-3') B2	Len	Tm	Sequence (5'-3') B3	Len	Tm
S1	ctgcaccaaaggtccaacc	19	60.9	acattctgtgtaactccaatacca	24	59.4
S2	aagtgattgtacccgctaacag	22	60	ccattaaacctataagccatttgc	24	58.1
S3	gctaacagtgcagaagtgtatt	22	58.3	ctgcaccaaaggtccaacc	19	60.9
S4	cctgcaccaaaggtccaac	19	60.9	ccattaaacctataagccatttgc	24	58.1
S5	acagggacttctgtgcagtt	20	61	ggagtaagttgatctgcatgaa	22	57.8
S6	aacagggacttctgtgcagt	20	61	ggagtaagttgatctgcatgaa	22	57.8
S7	cgctaacagtgcagaagtgtat	22	60.4	ctgcaccaaaggtccaacc	19	60.9

Primer sets #	Sequence (5'-3') FIP(F1c+F2)	Len	Sequence (5'-3') BIP(B1c+B2)	Len	PubMed Accession
S1	gcaaaggtggcatattgctgctagagacctcattt	35	tgattgctcaatacacctgcaccaaaggtccaacc	35	NC_045512
S2	aggccgttaaacttaatatggtgattgccttggtga	36	gttttgccacctttaagtgattgtacccgctaacag	36	NC_045512
S3	gaggtctctagcagagtgacacttgcagatgctg	34	gtttaacggccttacgctaacagtgcagaagtgtatt	37	NC_045512
S4	gcaaaggtggcaaacttggtgatattgctgctagag	36	tgattgctcaatacaccctgcaccaaaggtccaac	35	NC_045512
S5	gtgtctgtggatcaaagtttctgcctttccaacaat	36	acaccaggaacaaaacagggacttctgtgcagtt	34	NC_045512
S6	tcaagtgtctgtggctgcctttccaacaatttggc	35	caccaggaacaaataaacagggacttctgtgcagt	35	NC_045512
S7	tgaggtctctagcacacttgcagatgctggctt	33	tttaacggccttactcgctaacagtgcagaagtgtat	37	NC_045512

*** Note: Every primer set is composed of eight oligos, in which FIP is made by F1c+F2, and BIP is made by B1C+B2.**

Table S6. Case #16 RT-LAMP product sequencing results

**Primers to establish library**	**Forward**	AATGATACGGCGACCACCGAGATCTACACCTAGACTGCACACTCTTTCCCTACACGACGCTCTTCCGATCTGGGCGCGATCAAAACAACG		index in red	underscore incates the F2 of N27
	**Reverse**	CAAGCAGAAGACGGCATACGAGATCTAGACTGCGTGACTGGAGTTCAGACGTGTGCTCTTCCGATTCGGTAGTAGCCAATTTGGTC	**SARS-CoV-2 genomic RNA**	index in red	underscore incates the B2 of N27
**Sequencing results**		**Top 10 reads**	(MN908947.3), % Identities	**read count**	**% of total reads**
		TCGGCCCCAAGGTTTACCCAATAATACTGCGTCTTGGTTCACCGCTCTCACTCAACATGGCAAGGAAGACCTTAAATTCCCTCGAGGACAAGGCGTTCCAATTAAAACAAAAACCATACAAAATAACA	100%	1497377	38.33%
		TCGGCCCCAAGGTTTACCCAATAATACTGCGTCTTGGTTCACCGCTCTCACTCAACATGGCAAGGAAGACCTTAAATTCCCTCGAGGACAAGGCGTTCCAATTAACACCAATAGCAGTCCAGAT	100%	1193621	30.55%
		TCGGCCCCAAGGTTTACCCAATAATACTGCGTCTTGGTTCACCGCTCTCACTCAACATGGCAAGGAAGACCTTAAATTCCCTCGAGGAAAAGCCTTACAAATAAAAACAAAAACAATACAAAATAAAA	95%	182513	4.67%
		TCGCACCCAAGCTTAACCCAATAATACTCCGACTTGCTTCAACGCACTCACTCAACATGGCAAGGAAGACCTTAAATTCCCTCGAGGACAAGGCGTTCCAATTAACACCAATAGCAGTCCAGAT	94%	106275	2.72%
		ACCCAATAATACTGCGTCTTGGTTCACCGCTCTCACTCAACATGGCAAGGAAGACCTTAAATTCCCTCGAGGACAAGGCGTTCCAATTAACACCAATAGCAGTCCAGAT	100%	93284	2.39%
		TCGGCCCCAAGGTTTACCCAATAATACTGCGTCTTGGTTCACCGCTCTCACTCAACATGGCAAGGAAGACCTTAAATTCCCTCGAGGACAAGGCGTTCCAATTAACACCAA	100%	68159	1.74%
		TCGGCCCCAAGGTTTACCCAATAATACTGCGTCTTGGTTCACCGCTCTCACTCAACATGGCAAGGAAGACCTTAAATTCCCTCGAGGACAAGGCGTTCCAATTAACACCAATAGCAGTCCAGATGACC	100%	60954	1.56%
		TCGGCCCCAAGGTTTACCCAATAATACTGCGTCTTGGTTCACCGCTCTCACTCAACATGGCAAGGAAGACCTTAAATTCCCTCGAGGACAAGGC	94%	48579	1.24%
		CGGCCCCAAGGTTTACCCAATAATACTGCGTCTTGGTTCACCGCTCTCACTCAACATGGCAAGGAAGACCTTAAATTCCCTCGAGGACAAGGCGTTCCAATTAACACCAATAGCAGTCCAGAT	100%	33875	0.87%
		CGGCCCCAAGGTTTACCCAATAATACTGCGTCTTGGTTCACCGCTCTCACTCAACATGGCAAGGAAGACCTTAAATTCCCTCGAGGACAAGGCGTTCCAATTAA	100%	23569	0.60%
		**Total**		**3906852**	**84.68%**

Table S7. ACTB RT-LAMP primer set

**ACTB**	
**FIP**	GAGCCACACGCAGCTCATTGTATCACCAACTGGGACGACA
**BIP**	CTGAACCCCAAGGCCAACCGGCTGGGGTGTTGAAGGTC
**FL**	TGTGGTGCCAGATTTTCTCCA
**BL**	CGAGAAGATGACCCAGATCATGT
**F3**	AGTACCCCATCGAGCACG
**B3**	AGCCTGGATAGCAACGTACA

Reference

Zhang, Y., Ren, G., Buss, J., Barry, A. J., Patton, G. C., and Tanner, N. A. (2020)

Enhancing colorimetric loop-mediated isothermal amplification speed and sensitivity with guanidine chloride. Biotechniques 69, 178-185

Table S8. Real-time PCR primers

**E-Sarbeco***	**F**	ACAGGTACGTTAATAGTTAATAGCGT
	**R**	ATATTGCAGCAGTACGCACACA
**Beta-actin (ACTB)**	**F**	CTATCCCTGTACGCCTCTGG
	**R**	GTCACCGGAGTCCATCACC
**Human coronavirus 229E**	**F**	CGTGTTGGGACAGACATACTATC
	**R**	CTGAACACCTGAAGCCAATCTA
**Human coronavirus OC43**	**F**	TGGAGCGTATGGCTGATTTG
	**R**	GCATAGTTTGCAAGGCAGAAAC
**Influenza A virus (H1N1)**	**F**	GGGAACGGTGACCCAAATAA
	**R**	CAAGTGCACCAGCAGAATAAC
**Human respiratory syncytial virus**	**F**	GGCAGTAGAGTTGAAGGGATTT
	**R**	TGCACACTAGCATGTCCTAAC
**Human rhinovirus 16**	**F**	GTCAAATGGATCCAGCCTCA
	**R**	GGGTCTTGAGAGAAGTCCAATC

*Corman, V. M., Landt, O., Kaiser, M., Molenkamp, R., Meijer, A., Chu, D. K., Bleicker, T., Brunink, S., Schneider, J., Schmidt, M. L., Mulders, D. G., Haagmans, B. L., van der Veer, B., van den Brink, S., Wijsman, L., Goderski, G., Romette, J. L., Ellis, J., Zambon, M., Peiris, M., Goossens, H., Reusken, C., Koopmans, M. P., and Drosten, C. (2020) Detection of 2019 novel coronavirus (2019-nCoV) by real-time RT-PCR. Euro Surveill 25
