# Supplementary material for: Developing RT-LAMP assays for rapid diagnosis of SARS-CoV-2 in saliva
Source: eBioMedicine. 2021 Dec 16;75:103736. doi: 10.1016/j.ebiom.2021.103736 (PMC8674011; doi:10.1016/j.ebiom.2021.103736)
Supplement: Supplementary file 2 [file mmc2.pdf]

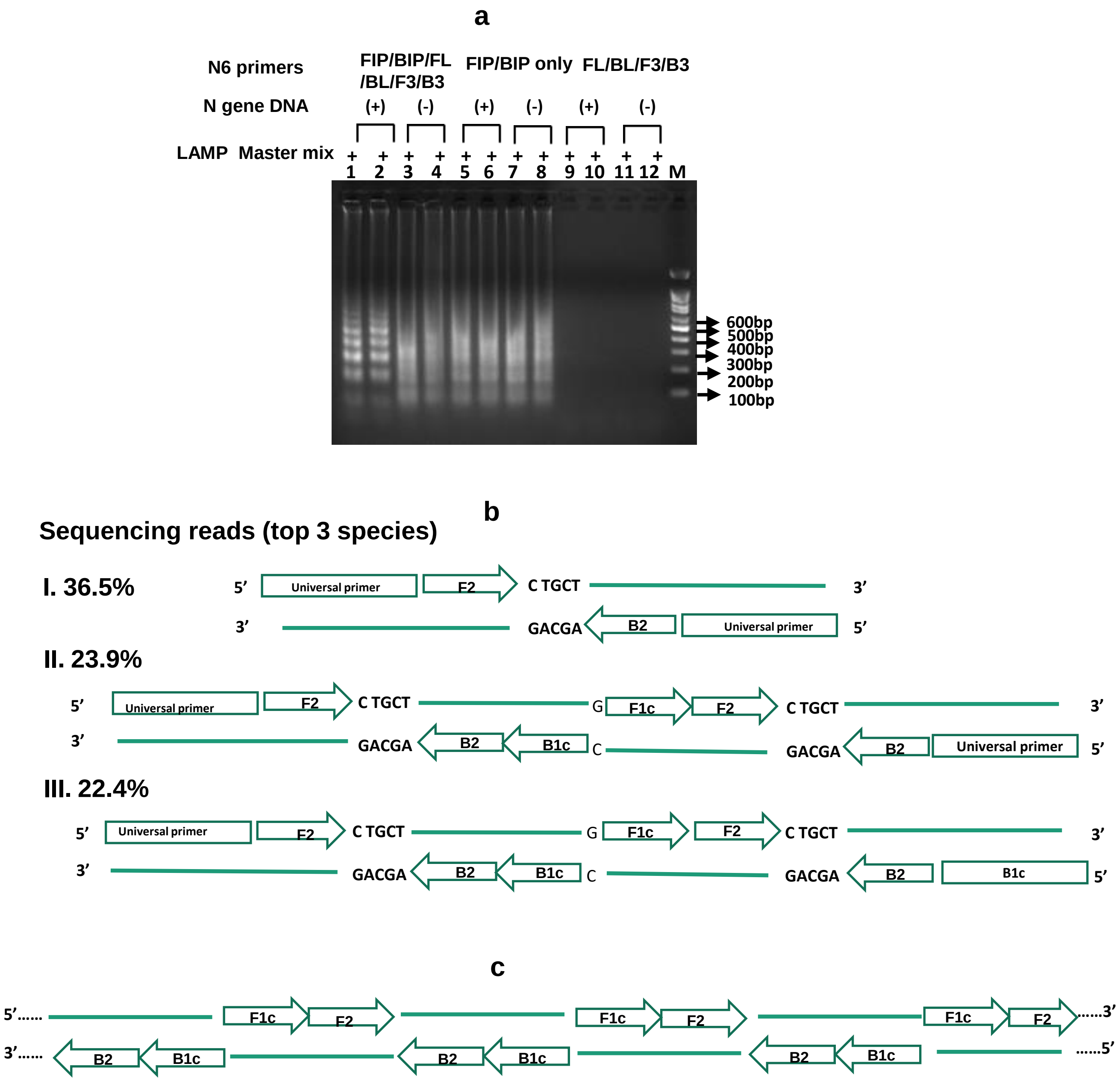

Figure S1. Analysis of the false-positive products from the N6 assay. a. Gel electrophoresis of the LAMP products from the reactions, in the presence or absence of the template DNA (the plasmid containing the N gene sequence of SARS-CoV-2) (lanes 1-12): in the presence of full set primers (lanes 1-4); in the presence FIP/BIP primers only (lanes 5-8), or in the presence of other primers only (reactions 9-12). **b.** Illumina sequencing analysis of the non-specific products as shown in lanes 3-8. **c.** Hypothesized model for the formation of non-specific LAMP products.

Target sequence

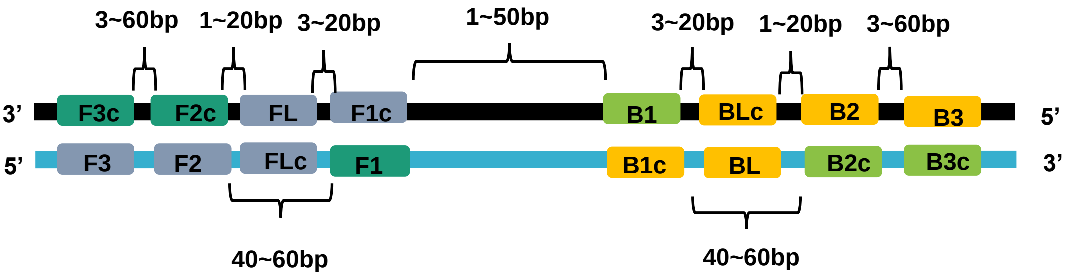

Primer Length

bp	F3	F2	FL	F1	B1	BL	B2	B3	FIP(F2+F1c)	BIP(B2+B1c)
Min	19	19	19	12	12	19	19	19		
Max	25	25	25	17	17	25	25	25	37	37
Opti	22	22	22			22	22	22		

Figure S2. Restraints on the relative position and length of the LAMP primers.

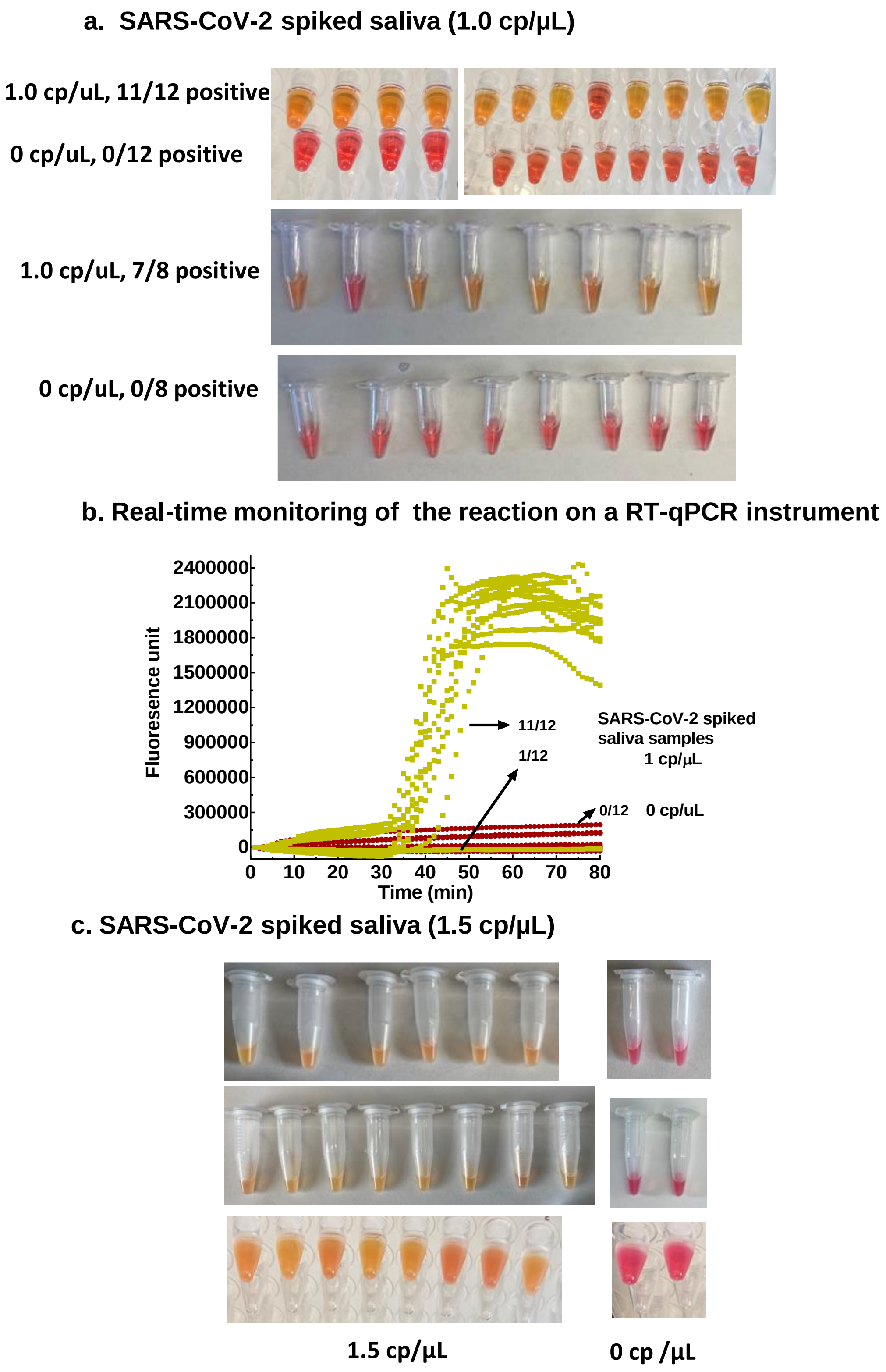

Figure S3. The sensitivity and specificity of the N27/N5 assay (120 µL reaction volume) assessed by colorimetric/fluorescence readout. a. Saliva samples spiked with 1.0 cp/µL SARS-CoV-2 particles. N27/M5 assays were performed either in 96-well PCR plate (0.2 mL) in a real-time PCR instrument (first panel of **a**) or in 0.5 mL Eppendorf tubes in a heating block (2nd & 3rd panels of **a**). **b.** Real-time monitoring of the spiked-in reactions as well as negative control reactions as shown in the first panel of **a**. **c.** Colorimetric readout of saliva samples (in 1.5 mL tubes or 96-well PCR plate) spiked with 1.5 cp/µL SARS-CoV-2 particles.

a. Colorimetric change at the end of reaction

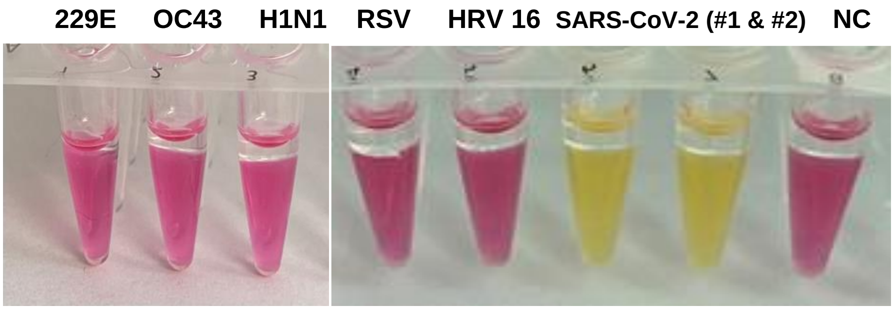

b. Real-time monitoring of the reaction on a real-time PCR instrument

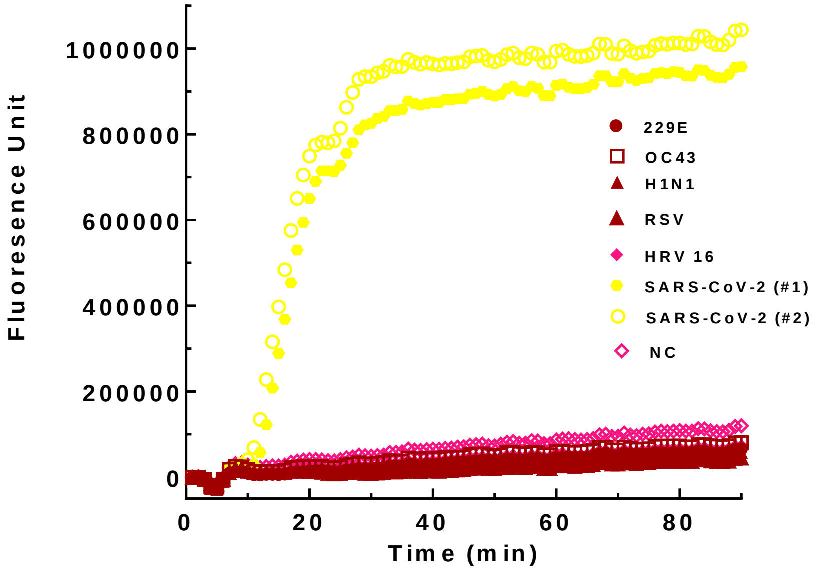

Figure S4. The cross-reactivity of the N27/M5 assay with other common respiratory viruses. Genomic RNAs (9 µL) from human coronavirus 229E (Ct ~23), human coronavirus OC43 (Ct ~23), influenza A virus (H1N1, Ct ~16), human respiratory syncytial virus (RSV) (Ct ~14), human rhinovirus 16 (HRV 16, Ct ~14), SARS-CoV-2 (Ct ~24 and ~25 for #1 and #2, respectively), and HeLa cell RNA (1 ng/µL, as negative control) were added to the N27/M5 RT-LAMP assay (120 µL volume). Assay positivity was defined by: (**a**) colorimetric changes at the end of the reaction, or (**b**) fluorescent signals detected by the real-time PCR instrument. The relative concentration of the genomic RNA from each virus was evaluated by standard RT-qPCR with ~0.5 µL RNA input, using primers specific to each viral genome sequence (Supplementary Table S8). SARS-CoV-2 RNA (samples #1 and #2) were isolated from 300 µL saliva of healthy subjects spiked with 6,000 cp/µL and 3,000 cp/µL heat-inactivated SARS-CoV-2, respectively. The results shown here represent three independent measurements with similar findings. NC: negative control.
