# Supplementary material for: Developing RT-LAMP assays for rapid diagnosis of SARS-CoV-2 in saliva
Source: eBioMedicine. 2021 Dec 16;75:103736. doi: 10.1016/j.ebiom.2021.103736 (PMC8674011; doi:10.1016/j.ebiom.2021.103736)
Supplement: Supplementary file 3 [file mmc3.docx]

**Supplementary materials caption:**

Table S1. N gene primer sets

Table S2. N6 assay false-positive product sequencing

Table S3. The loop stem (F1c and B1c), LAMP amplification efficiency, and specificity

Table S4. The M gene primer sets

Table S5. The S gene primer sets

Table S6. Case #16 RT-LAMP product sequencing results

Table S7. ACTB RT-LAMP primer set

Table S8. Real-time PCR primers

Figure S1. Analysis of the false-positive products from the N6 assay.

Figure S2. Restraints on the relative position and length of the LAMP primers.

Figure S3. The sensitivity and specificity of the N27/N5 assay (120 µL reaction volume) assessed by colorimetric/fluorescence readout.

Figure S4. The cross-reactivity of N27/M5 assay with other common respiratory viruses.

Reagent Validation:

Validation 1. HeLa cell line authentication: short tandem repeat (STR) profile against the ATCC Human Cell STR Database

Validation 2. SiHa cell line authentication: short tandem repeat (STR) profile against the ATCC Human Cell STR Database
